# Enhanced Growth and Contrasting Effects on Arsenic Phytoextraction in *Pteris vittata* through Rhizosphere Bacterial Inoculations

**DOI:** 10.3390/plants13152030

**Published:** 2024-07-24

**Authors:** Maria Luisa Antenozio, Gianluigi Giannelli, Rosaria Fragni, Diego Baragaño, Patrizia Brunetti, Giovanna Visioli, Maura Cardarelli

**Affiliations:** 1 IBPM-CNR c/o Dipartimento di Biologia e Biotecnologie, Sapienza Università di Roma, Piazzale Aldo Moro, 00185 Roma, Italy; marialuisaantenozio@gmail.com (M.L.A.); maura.cardarelli@gmail.com (M.C.); 2 Department of Chemistry, Life Sciences and Environmental Sustainability, University of Parma, Parco Area delle Scienze 11/A, 43124 Parma, Italy; gianluigi.giannelli@unipr.it; 3 SSICA, Experimental Station for the Food Preserving Industry, viale Tanara 31a, 43121 Parma, Italy; Rosaria.Fragni@ssica.it; 4 Instituto de Ciencia y Tecnologia del Carbono, INCAR-CSIC, Francisco Pintado Fe 26, 33011 Oviedo, Spain; diego.baragano@incar.csic.es

**Keywords:** phytoextraction, arsenic, *Pteris vittata*, rhizosphere bacteria

## Abstract

This greenhouse study evaluated the effects of soil enrichment with *Pteris vittata* rhizosphere bacteria on the growth and accumulation of arsenic in *P. vittata* grown on a naturally As-rich soil. Inoculations were performed with a consortium of six bacteria resistant to 100 mM arsenate and effects were compared to those obtained on the sterilized soil. Selected bacteria from the consortium were also utilized individually: PVr_9 homologous to *Agrobacterium radiobacter* that produces IAA and siderophores and shows ACC deaminase activity, PVr_15 homologous to *Acinetobacter schindleri* that contains the arsenate reductase gene, and PVr_5 homologous to *Paenarthrobacter ureafaciens* that possesses all traits from both PVr_9 and PVr_15. Frond and root biomass significantly increased in ferns inoculated with the consortium only on non-sterilized soil. A greater increase was obtained with PVr_9 alone, while only an increased root length was found in those inoculated with either PVr_5 or PVr_15. Arsenic content significantly decreased only in ferns inoculated with PVr_9 while it increased in those inoculated with PVr_5 and PVr_15. In conclusion, inoculations with the consortium and PVr_9 alone increase plant biomass, but no increase in As phytoextraction occurs with the consortium and even a reduction is seen with PVr_9 alone. Conversely, inoculations with PVr_5 and PVr_15 have the capacity of increasing As phytoextraction.

## 1. Introduction

Arsenic (As) is one of the most toxic elements in the ecosystem released through natural processes and anthropogenic activities such as the use of fossil fuels and biomass burning, mining, industrial activities, and the use of arsenical pesticides in agriculture [1]. Being non-essential and toxic, its uptake in plants results in morphological, physiological, and biochemical alterations, as well as genotoxicity (e.g., defoliation, root shortening, impaired photosynthesis, impaired efficiency of Calvin cycle enzymes, oxidative stress, etc.), that ultimately result in restrictions in plant growth, development, and productivity [2,3].

The most common As inorganic compounds, arsenate As(V) and arsenite As(III), can be absorbed by crop plants and spread throughout the food chain, becoming an issue not only for plant growth, but also for human health [4].

The presence of As in the environment has led to the development of several strategies for removing this pollutant from contaminated sites. Phytoextraction is an environmentally friendly method that uses the ability of plants to remove elemental pollutants from soil and waters. However, the efficiency of phytoextraction depends on plants’ tolerance to the specific element and their ability to accumulate it. For this reason, hyperaccumulator plants that can tolerate high concentrations of As, extract As from the soil, and concentrate it in the biomass above the soil have been mainly used for As phytoextraction. Among As hyperaccumulators, *Pteris vittata* L. (*P. vittata*—Chinese brake fern) is optimal for phytoextraction due to its ability to tolerate soils containing as much as 1500 p.p.m. As, to take up large amounts of As in a short time, and to concentrate > 2.3% (W/W) of As in frond dry matter [5].

However, effective phytoextraction also depends on Plant Growth-Promoting Rhizobacteria (PGPR) that colonize the rhizosphere. Microbes form symbiotic relationships with plants and can enhance their growth and As availability [6]. The combined use of plants and microbes is also known as phytobial remediation [6,7]. 

PGPR adopt different strategies to cope with As contamination in the soil: (i) modulating phytohormone levels, mainly through the production of auxin (IAA), or decreasing ethylene levels via the biosynthesis of 1-aminocyclopropane-1-carboxylate (ACC) deaminase; (ii) increasing mineral content by means of siderophores, phosphate solubilization and nitrogen fixation [6,7]; and (iii) mediating the conversion between the oxidized and reduced states of As. In particular, As(V)-reducing bacteria, containing the *arsC* gene, promote the growth and As-accumulating capacity of *P. vittata* by improving the bioavailability of As in the soil [8,9]. The dominant bacterial genera associated with the rhizosphere of *P. vittata* grown in As-contaminated soils are *Bacillus*, *Lysinibacillus*, *Acinetobacter*, *Arthrobacter*, *Pseudomonas*, *Agrobacterium*, and *Ochrobactrum* [8,10,11].

On the other hand, *P. vittata* is able to produce radical exudates such as malic acid and pteroids that are able to support the growth of rhizobacteria and most importantly select functional bacteria and influence phytoextraction efficiency [12,13,14]. 

Current findings suggest that As-resistant bacteria induce an increased phytoextraction efficiency of *P. vittata* often associated with increased plant growth. Yang et al. [15] reported that, probably by facilitating As(III) oxidation and absorption, the inoculation of *Cupriavidus basilensis* increased As accumulation in *P. vittata* aboveground biomass up to 171%. Feng et al. [16] found that an As(V)-reducing *Pseudomonas* strain greatly increased *P. vittata* growth and As content both in aboveground and belowground biomass. Lampis et al. [10] reported an increase by 4-fold in the bioconcentration factor (BCF) in fronds of *P. vittata* inoculated with a mixture of five bacterial strains including *Bacillus* sp. MPV12, *Delftia* sp. P2III5, *Pseudomonas* sp. P1III2, *Pseudoxanthomonas* sp. P4V6, and *Variovorax* sp. P4III4. Moreover, they showed increased plant biomass. More recently Li et al. [17] showed that inoculations with *Enterobacter* sp. E1 increased arsenic uptake in *P. vittata* by promoting plant growth. In contrast Yang et al. [18], in a 3-year field trial experiment, performed a single *P. vancouverensis* inoculation which resulted in an increase in As accumulation of 48%, 54%, and 35% in *P. vittata* plants, which, however, is not associated, with increased plant biomass. Similarly, Abou-Shanab et al. [19] showed that inoculations with *P. monteilii*, *P. plecoglossicida*, *O. intermedium* strains, and *A. tumefaciens* strains MK344655, MK346994, and MK346997 significantly increased As uptake in *P. vittata* plants, while it had no effects on plant biomass. In addition, Yang et al. [20] investigated the metabolomic correlation of *P. vittata* and associated rhizospheric microorganisms during As phytoextraction and suggested that rhizospheric plant–microbes have synergistic effects with hyperaccumulators on phytoextraction.

However, several trials have been carried out with single or a few bacterial strain inocula on sterilized soils or hydroponically culture [8] or soils amended with different concentrations of As, thus in the absence of a specific microbiome [19]. Therefore, it is unclear whether the bacteria maintain their capabilities of increasing As phytoextraction efficiency and/or plant biomass in a natural soil colonized by high numbers of resident rhizobacteria.

In a previous work, we isolated sixteen As(V)-tolerant bacterial strains from the rhizosphere of *P. vittata* grown on a naturally As-contaminated soil (named Bagnaccio) with a high As content near Viterbo in the Lazio region, central Italy. This site is a naturally As-rich volcanic area, and the As concentration in the soil is not due to anthropogenic contamination. Bagnaccio soil has been previously characterized as a calcareous soil with an average As concentration of 750.11 mg kg^−1^, of which 28% is bioavailable [11]. Only two isolates have been previously associated with As and six out of the sixteen bacteria were found to be resistant up to 100 mM As(V). Among them, two belong to the *Bacillus* genus (PVr_2 and PVr_17), previously shown to be abundant in As-contaminated soils and waters [21]. Another bacterial isolate shows homology with the genus *Agrobacterium* (PVr_9), also found in *P. vittata* rhizosphere, showing resistance to As(III) [11,22] and/or capable of performing both As oxidation and reduction. Other isolates, *Paenantrobacter urefaciens* (*P. urefaciens*) (PVr_5) and *Acinetobacter schindleri* (*A. schindleri*) (PVr_15 and PVr_16), have never been previously associated with *P. vittata* roots. The selected consortium bacteria possess multiple PGPR beneficial traits. 

The objective of this study was to assess the effects of these highly As-resistant rhizobacteria on plant growth in relation to As accumulation and phytoextraction, in the presence or absence of resident bacteria. To this aim, *P. vittata* plants were grown on sterilized and non-sterilized high-As soil from which the rhizobacteria were isolated and were then inoculated with the consortium of six bacteria and subsequently with individual bacteria from the consortium. 

## 2. Results

### 2.1. Plants Grown on Natural Bagnaccio Soil Inoculated with the Whole Consortium Show Increased Biomass

**Figure 1:**
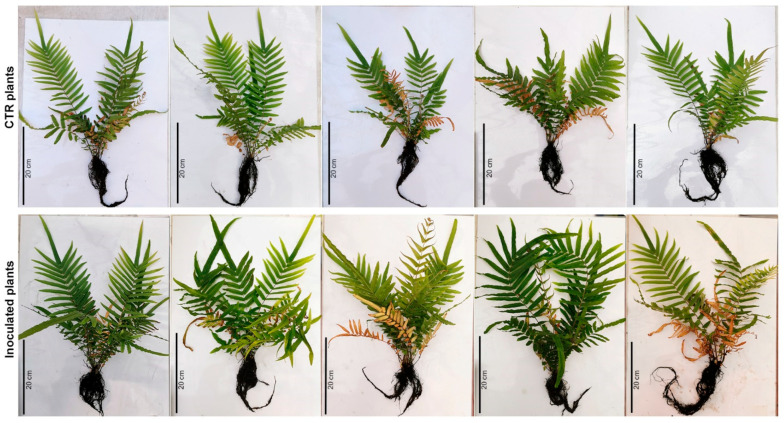
*P. vittata* plants inoculated with the consortium on non-sterile soil at the end of the experiment. CTR plants (control uninoculated ferns) are in the upper panel and inoculated ferns are in the lower panel.

To determine whether Bagnaccio soil enriched with highly As-resistant rhizobacteria influences fern growth and accumulation of As, ten 6-month-old *P. vittata* plants were transferred into Bagnaccio soil under greenhouse conditions in non-sterile soil (Figure 1). Then, the inoculum with the whole consortium (PVr_2, PVr_5, PVr_9, PVr_15, PVr_16, PVr_17) was applied to five plants at the beginning of the experiment and after 2 months [10].

**Figure 2:**
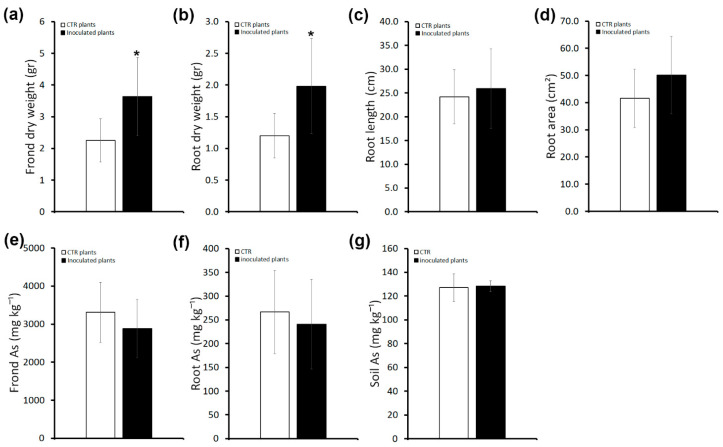
Effects of inoculums with the consortium in *P. vittata* plants grown on non-sterile soil on plant growth, As accumulatio and phytoextraction efficiency. (**a**) *P. vittata* frond biomass (gr dry weight). (**b**) *P. vittata* root biomass (gr dry weight). (**c**) *P. vittata* root length (cm). (**d**) *P. vittata* root area (cm^2^). (**e**) Arsenic concentration (mg kg^−1^ dry weight) in *P. vittata* fronds. (**f**) Arsenic concentration (mg kg^−1^ dry weight) in *P. vittata* roots. (**g**) Arsenic concentration (mg kg^−1^) in soil at the end of the experiment. Data are expressed as a mean value (n = 5). Error bars indicate the standard error (SE). Asterisk indicates a significant difference from the non-inoculated control value (CTR): * *p* < 0.05.

*P. vittata* frond and root biomass (dry weight) and root length and area were analyzed two months after the last inoculum (Figure 2a–d). As shown in Figure 2a,b, in inoculated plants, a significant increase in frond and root biomass (~61% and ~65%, respectively) was observed. Conversely, root length and area were comparable between inoculated and uninoculated plants (Figure 2c,d).

To compare the amount of As, a quantitative analysis by ICP-OES was performed in roots and fronds of inoculated and uninoculated plants. As shown in Figure 2e,f, As content in inoculated plants as well as frond BAF and root BAF values (Table S1) were comparable to uninoculated plants. In agreement, the soil As content of inoculated plants, as assessed at the end of the experiment, was the same as that of uninoculated plants (Figure 2g).

These results indicate a plant-biomass-promoting effect of the bacterial consortium that does not have an impact on the accumulation of As.

**Figure 3:**
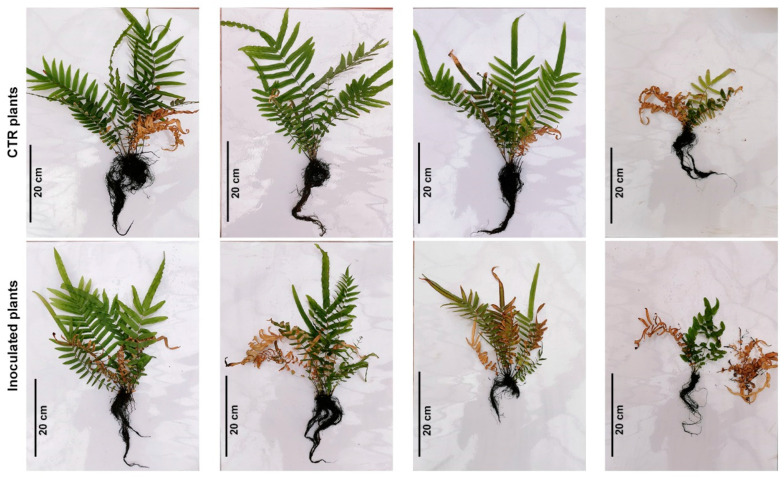
*P. vittata* plants inoculated with the consortium on sterile soil at the end of the experiment. CTR plants (control uninoculated ferns) are in the upper panel and inoculated ferns are in the lower panel.

To compare the effects of Bagnaccio soil enrichment with highly As-resistant rhizobacteria to that of inoculations in the absence of/with a reduced resident bacterial community, eight 6-month-old *P. vittata* plants were transferred into sterilized Bagnaccio soil under greenhouse conditions. The inoculums with the whole consortium (PVr_2, PVr_5, PVr_9, PVr_15, PVr_16, PVr_17) were applied as before at the beginning of the experiment and after 2 months (Figure 3). 

**Figure 4:**
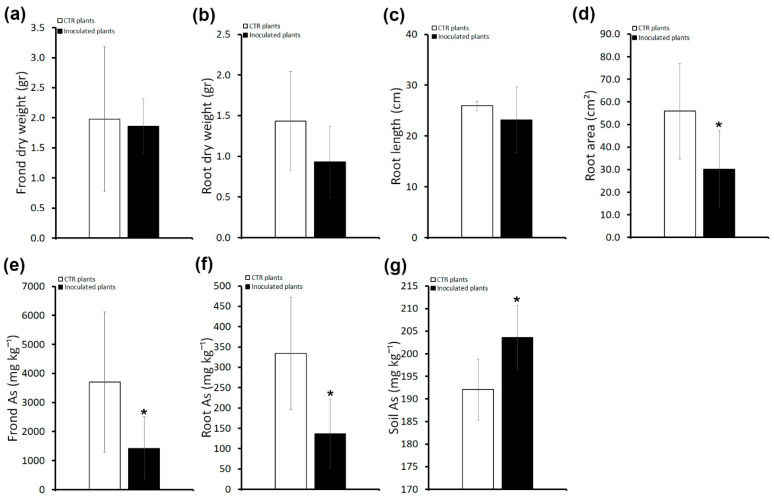
Effects of inoculums with the consortium in *P. vittata* plants grown on sterile soil on plant growth, As accumulation, and phytoextraction efficiency. (**a**) *P. vittata* frond biomass (gr dry weight). (**b**) *P. vittata* root biomass (gr dry weight). (**c**) *P. vittata* root length (cm). (**d**) *P. vittata* root area (cm^2^). (**e**) Arsenic concentration (mg kg^−1^ dry weight) in *P. vittata* fronds. (**f**) Arsenic concentration (mg kg^−1^ dry weight) in *P. vittata* roots. (**g**) Arsenic concentration (mg kg^−1^) in soil at the end of phytoextraction. Data are expressed as a mean value (n = 4). Error bars indicate the standard error (SE). Asterisk indicates a significant difference from the non-inoculated control value (CTR): * *p* < 0.05.

*P. vittata* frond and root biomass (dry weight) and root length and area were analyzed two months after the last inoculum. Although all plants grew slightly worse (Figure 3), there was no significant difference between the biomass of both fronds and roots of uninoculated plants with those grown on unsterilized soil (Figure 4a,b compared to Figure 2a,b). Conversely, inoculated plants showed a significant reduction in root area compared with uninoculated plants grown on soil with or without sterilization (Figure 4d). Moreover, while, in uninoculated plants, As content is comparable to that of uninoculated plants grown on unsterilized soil, quite unexpectedly, it is significantly lower in inoculated plants than in all control plants (Figure 2e,f and Figure 4e,f). Consequently, we found that the frond BAF and root BAF values were low in inoculated plants (Table S2). In agreement, the As content in the soil surrounding the inoculated plants, as assessed at the end of the experiment, was higher than that of uninoculated plants (Figure 4g).

These results indicate that in the absence of/reduction in resident bacteria, inoculation with consortium bacteria not only does not promote plant growth, but on the contrary inhibits root system development with a negative impact on As accumulation. 

Thus, due to these findings, subsequent experiments were performed without soil sterilization.

### 2.2. Inoculums of Individual Bacterial Strains Promote P. vittata Growth Differently and Have Opposite Effects on As Phytoextraction

To assess whether individual and selected bacteria influence fern growth and accumulation of As, eight 6-month-old *P. vittata* plants were transferred into Bagnaccio soil and inoculated with three individual bacterial strains showing high As(V) resistance: PVr_5 and PVr_9, which produce siderophore and IAA and show ACC deaminase activity, and PVr_15, which has no PGP traits but is highly resistant to As(V) [11]. *P. vittata* frond and root biomass and root length and area were analyzed two months after the last inoculum.

**Figure 5:**
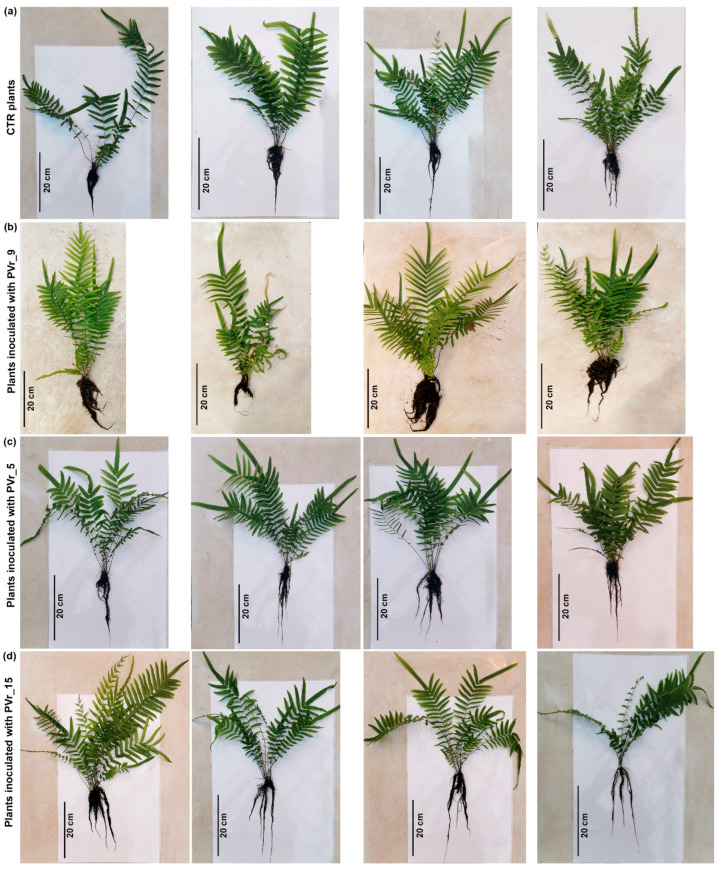
(**a**) Uninoculated *P. vittata* plants (CTR, i.e., control), (**b**) plants inoculated with PVr_9 strain, (**c**) plants inoculated with PVr_5 strain, and (**d**) plants inoculated with PVr_15 strain on non-sterile soil at the end of the experiment.

**Figure 6:**
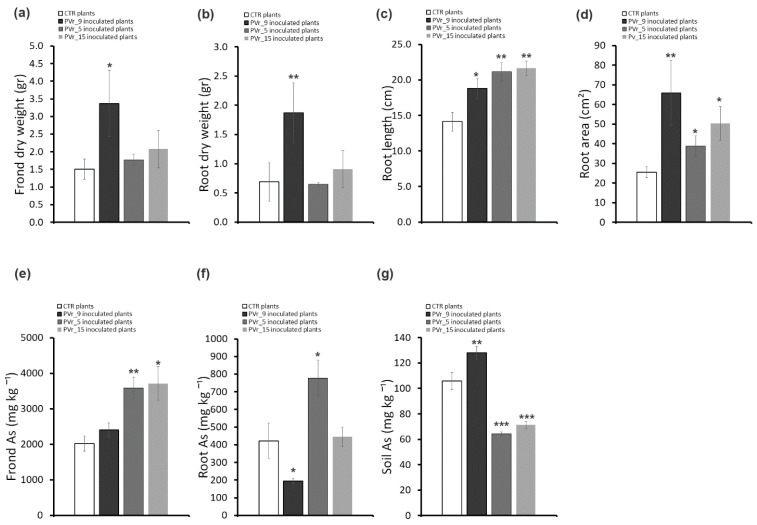
Effects of inoculums with PVr_9, PVr_5 and PVr_15 on *P. vittata* plant growth, As accumulation, and phytoextraction efficiency. (**a**) *P. vittata* frond biomass (gr dry weight). (**b**) *P. vittata* root biomass (gr dry weight). (**c**) *P. vittata* root length (cm). (**d**) *P. vittata* root area (cm^2^). (**e**) Arsenic concentration (mg kg^−1^ dry weight) in *P. vittata* fronds. (**f**) Arsenic concentration (mg kg^−1^ dry weight) in *P. vittata* roots. (**g**) Arsenic concentration (mg kg^−1^) in soil at the end of phytoextraction. Data are expressed as a mean value (*n* = 4). Error bars indicate the standard error (SE). Asterisk indicates a significant difference from the non-inoculated control value (CTR): * *p* < 0.05, ** *p* < 0.01, *** *p* < 0.001.

As shown in Figure 5 and Figure 6a,b in plants inoculated with PVr_9, a significant increase in frond and root biomass, as well as a slight increase in root length and a robust increase in root area, was observed (Figure 5 and Figure 6c,d). 

Conversely, in ferns inoculated with PVr_5 or PVr_15, frond and root biomass was comparable to that of uninoculated plants (Figure 5 and Figure 6a,b), while an increase in root length and in root area was observed, but lower than that of PVr_9 (Figure 5 and Figure 6c,d). 

These data show that PVr_9 alone can stimulate plant growth, while a more modest effect is obtained on only root growth by inoculations with PVr_5 or PVr_15 isolates. 

Notably, As content in plants inoculated with PVr_9 is lower in roots and unchanged in fronds compared with non-inoculated plants (Figure 6e,f). In agreement, the TF, frond BAF (Table S3), and As content in soil (Figure 6g) are higher compared with those of uninoculated plants, while root BAF is lower than that of uninoculated plants (Table S3).

Conversely, As content is significantly higher in fronds and roots of plants inoculated with PVr_5 and only in fronds of those inoculated with PVr_15 compared with uninoculated plants (Figure 6e,f). In agreement, we found a slightly increased TF and high values of frond BAF and root BAF in plants inoculated with PVr_15, while As content in the soil was lower compared with uninoculated plants. On the other hand, we found high values only of frond BAF in plants inoculated with PVr_5, while As content in the soil was lower compared with uninoculated plants (Table S3). 

**Figure 7:**
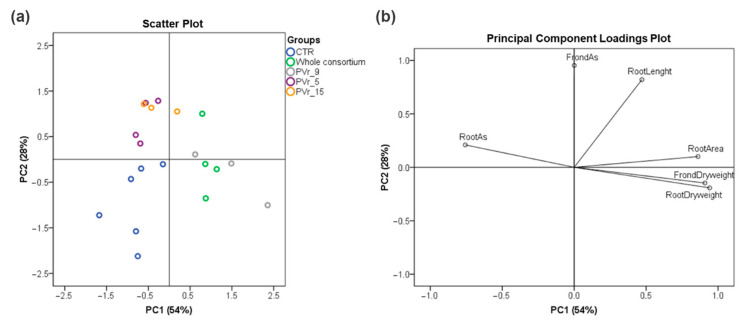
Principal component analysis (PCA) with PC1 and PC2 for treatment classification using the variables examined in the ferns. Variance proportions are shown along each component axis. (**a**) Scattered plot. (**b**) Loadings plot. Treatments: control (CNT); ferns inoculated with six As-resistant strains (whole consortium); PVr_9-inoculated ferns (PVr_9); PVr_5-inoculated ferns (PVr_5); PVr_15-inoculated ferns (PVr_15).

PCA performed on the analyzed fern traits revealed a clear discrimination between groups (Figure 7a). PC1 explained the largest variability and allowed for separating uninoculated ferns (CRT) from ferns inoculated with the consortium of six As-resistant strains (whole consortium) and ferns inoculated with the single PVr_9 strain (Figure 7a). The PC loading plot indicates that inoculated plants possessed higher root and frond dry weight and root area and lower As content in the roots with respect to uninoculated ones (Figure 7b). PC2 allowed for an efficient separation of PVr_5- and PVr_15-inoculated ferns from uninoculated ones (CRT), with PVr_5 and PVr_15 presenting longer roots and a higher content of As in fronds (Figure 7a,b). 

These results indicate that PVr_9 not only increases plant biomass, root growth, and As translocation, but quite unexpectedly reduces As accumulation in roots, while PVr_5 and PVr_15 promote only root development and increase As accumulation. 

## 3. Discussion

In this work, based on the growth of *P. vittata* plants inoculated with a consortium of six highly As(V)-resistant bacteria on unsterilized As-contaminated soil [11], we demonstrated that these bacterial strains promote plant growth but have no effect on the accumulation of As. Inoculations with the PVr_9 bacterial isolate alone increased fern biomass and notably reduced As accumulation, while those with the two different bacterial strains PVr_5 or PVr_15 alone promoted fern root growth and increased As accumulation and, therefore, phytoextraction efficiency.

We grew *P. vittata* plants on unsterilized As-rich Bagnaccio soil, located in the Viterbo region, central Italy [11], which contains resident bacteria and mimics the real situation in field experiments. *P. vittata* plants were inoculated with a consortium of six selected highly As(V)-resistant (up to 100 mM) bacterial strains isolated from the rhizosphere of *P. vittata* plants grown on Bagnaccio soil. 

We also performed individual inoculations with three selected bacteria from the consortium; PVr_9, the largest producer of IAA and siderophores with ACC deaminase activity; PVr_5, which produces (to a lesser extent) IAA and siderophores, has ACC deaminase activity, and also contains the *arsC* gene; and PVr_15, which only contains the *arsC* gene and does not show any of the tested PGPR traits. 

We found that soil enrichment, performed by inoculations with the whole consortium of As-resistant bacteria in non-sterilized soil, led to a promotion of plant growth as shown by the increased frond and root biomass (Figure 1 and Figure 2a,b). However, this effect appears to require the interaction of As-resistant bacteria with other *P. vittata* Bagnaccio soil bacteria, because inoculations on sterilized soil that are thus devoid of resident bacteria have a negative effect on fern growth. In fact, we showed that inoculated plants grown on sterilized soil have a significant reduction in root area compared with those grown on non-sterilized soil (Figure 4d). In addition, a slight negative effect on plant growth was also observed in uninoculated plants, possibly because of both the elimination of resident bacteria and the physiochemical changes in the soil caused by autoclaving. In agreement, it has been proposed that plant–microbe combinations assessed using spiked and/or sterilized soils do not usually reflect the in situ conditions, and therefore can lead to results different from those under field conditions [23]. This can also be related to the capacity of plants to select functional bacterial strains through the production of root exudates. Thus, microbial diversity and ecosystem functioning appeared to be strictly correlated [24]. In fact, some studies have shown that specialized functions in an ecosystem are dependent on microbial diversity and require specific taxonomic groups as well as particular metabolic pathways [25,26]. Thus, sterilization-induced destruction of the soil microbial community may impact plant capacities.

As for changes in soil properties such as soil acidity, electrical conductivity, cation and exchange capacity, these are known to be modified by autoclaving [27,28,29].

Unexpectedly, we found that the increased biomass in plants inoculated with the consortium of selected bacterial strains and grown on enriched soil is not associated with an increase in As accumulation. In support of this, the concentration of As in the soil is similar between inoculated and uninoculated plants at the end of the experiment. This may be due to interaction of consortium isolates with resident bacteria or to different properties of individual bacteria in the consortium (see below). Our findings are in contrast with most published data, which report increased As accumulation often associated with increased biomass in plants inoculated with PGPR bacteria [10] and indicate that these two features are not directly associated. 

Notably, by enriching the soil with the three different selected strains of the consortium separately, we identified PVr_9, which, to our knowledge, is the first isolate capable of reducing As phytoextraction while stimulating *P. vittata* growth as well as As translocation. In fact, we showed that PVr_9, which is homologous to *Agrobacterium radiobacter,* is able to increase frond and root biomass alone more than the consortium and to trigger root development by substantial lateral root production. The enhanced fern biomass and root growth exerted by PVr_9 are possibly related to the high IAA production of this strain and to the ACC deaminase activity that has already been associated with rhizobacteria such as *Bacillus* sp. MPV12, *Variovorax* sp. P4III4, and *Pseudoxanthomonas* [10]. In addition, PVr_9’s plant growth effects are not limited to the hyperaccumulator *P. vittata*, as increased root length and density as well as shoot area were observed in the model plant *Arabidopsis thaliana* inoculated with PVr_9 [30,31]. In terms of As accumulation in *P. vittata*, we found a significant decrease in As content in roots and an increase in As translocation in fronds (2.6-fold compared to CTR plants). As a result, PVr_9-inoculated plants showed a significant reduction in total As accumulation, which is consistent with an increase in As in the soil compared with uninoculated plants at the end of the experiment (Figure 6g). The effects of this bacterial isolate on As translocation could be due to the production of siderophores as previously shown in *Pteris cretica* [32]. However, in the latter, the siderophore-induced increase in As translocation was also associated with increased As uptake. A possible explanation for the reduction in As uptake exerted by PVr_9 in *P. vittata* can be related to the ability of this strain to form biofilms, which are known to form a protective barrier for roots against the uptake of As and other metals [33]. In agreement, it has been shown that As can induce bacterial exopolysaccharide (EPS) production in biofilm, and *Thiomonas* sp. CB2 was reported to trap As ions in its biofilm EPS in response to arsenic stress [34]. Similarly, Tournay et al. [35] showed that the endophyte *Rahnella laticis* PD12R synthesizes high volumes of EPS in response to As and sequesters As in the EPS. In addition, the growth of *A. thaliana* exposed to As was increased by inoculations with the *Pseudomonas* PD9R strain, while in vitro absorption of As into the biofilm of plant-associated bacteria was also observed for *Pseudomonas koreensis* AGB-1, *Kokuria flava* AB402, and *Bacillus viernamensis* AB403 [36,37]. Furthermore, biofilm formation and the C-OH and P=O polysaccharide groups were found to have a key role in the absorption of heavy metals such as Cu^2+^, Zn^2+^, Cd^2+^, and Pb^2+^ [38]. By single inoculations, we also found a limited effect on plant growth of PVr_5, which only increases root growth; this may be because of the lower production of IAA and siderophores compared to PVr_9. In partial agreement, while no growth-enhancing effect following inoculation with PVr_5 was observed in *A. thaliana* in the absence of As [30], a significant number of lateral roots were obtained with respect to uninoculated ones following treatment with 75 mM of As(V) (Giannelli et al., personal communication). The root-growth-stimulating effect on *P. vittata* can be due to the ACC deaminase activity of PVr_5, which can enhance root elongation, as previously demonstrated by other authors [39,40]. In addition, it can also be related to the reduction of As(V) to As(III) due to the bacterial *arsC* gene. Similarly, PVr_15, which also contains the *arsC* gene but has no ACC deaminase activity, showed increased root length. In terms of As accumulation, we found that PVr_5 is capable of increasing As content in both fronds and roots, while PVr_15 can increase As content only in fronds, possibly due to the slight increase in As translocation compared to uninoculated plants. These findings are also supported by the decrease in As in the soil compared with uninoculated plants at the end of the experiment. While, in plants inoculated with PVr_5, this could be the combined effect of siderophore production and of the reduction of As(V) to As(III), in the case of PVr_15, this might be only the result of As(V) reduction, as this isolate does not produce siderophores. Indeed, it is known that As(V) reduction can promote As uptake by *P. vittata* [9]. More work will be necessary to understand the mechanism by which these bacterial strains increase As phytoextraction. Moreover, inoculations with individual isolates revealed the presence of strains with opposite characteristics regarding As accumulation in the consortium and thus may explain why the consortium has no general effects on phytoextraction efficiency. Furthermore, the isolation of a strain that produces biofilms, possibly reducing As uptake, may be a consequence of the higher amount of As present in Bagnaccio soil compared with that in most soils used in many field trials [10,15,18]. Future work using different soils with high As concentration will be needed to clarify this point.

## 4. Materials and Methods

### 4.1. Site Description

Viterbo is a naturally As-rich volcanic area. Arsenic abundance and mobilization in this zone are a result of hydrothermal processes that cause the up-flow of thermal waters. Soil samples were collected from Bagnaccio, an area situated in the western side of Viterbo (Lazio, Italy) (42°27′30.4″ N 12°03′55.9″ E). Bagnaccio is a calcareous soil characterized by an average As concentration of 750.11 mg kg^−1^, of which 28% is bioavailable. Arsenic is present in its inorganic pentavalent form As(V) [11].

### 4.2. Bacterial Strains and Traits

Sixteen As(V)-tolerant bacterial strains were previously isolated from the rhizosphere of *P. vittata* plants grown in Bagnaccio soil and classified by 16S rDNA sequencing as reported by Antenozio et al. [11].

The ability of each bacterial strain to tolerate As(V) (1, 3, 6, 10, 75, 100 mM), produce siderophores, produce indole acetic acid (IAA) and ACC deaminase (an enzyme responsible for ethylene production), and contain the *arsC* gene coding for an As(V) reductase was assessed as previously described [11]. 

Six bacterial strains that were able to grow on As(V) up to 100 mM (PVr_2 (MT013508) homologous to *Bacillus simplex*, PVr_5 (MT013510) homologous to *Paenarthrobacter ureafaciens*, PVr_9 (MT013514) homologous to *Beijerinckia fuminensis* and recently reclassified as an *Agrobacterium radiobacter* strain by NCBI database, PVr_15 (MT013520) homologous to *Acinetobacter schindleri*, PVr_16 (MT013521) homologous to *Acinetobacter schindleri*, and PVr_17 (MT013522) homologous to *Bacillus halosaccharovorans* of the above 16 strains) were tested in this work as a part of a phytoremediation experiment with *P. vittata* plants for their ability to enhance biomass production and As phytoextraction from the soil.

### 4.3. Plant Cultivation and Inoculum Preparation

Bacterial strains were grown in 100 mL of sterilized LB broth medium [41,42]. Cultures were incubated at 28 °C, with shaking at 160 rpm, until reaching the absorbance at 600 nm of 0.2 (equivalent to approximately 1 × 10^8^ CFU mL^−1^), and were used for plant inoculation [10].

The propagation and growth of ferns were performed in the greenhouse under controlled conditions [43,44,45]. 

Six-month-old ferns were then transplanted into plastic pots (20 cm diameter, 14 cm high) containing 2 kg of sterilized and unsterilized Bagnaccio soil. A total of 100 mL of inoculum at a concentration of 10^8^ CFU/mL was inoculated in the soil around the rhizosphere zone. Frond, root, and soil As contents as well as frond and root dry weights and root length and area were evaluated with five different treatments (five or four replicates each): (i) non-inoculated plants; (ii) plants inoculated with all six bacterial strains (PVr_2, PVr_5, PVr_9, PVr_15, PVr_16, PVr_17) resistant to 100 mM As(V) [11]; (iii) plants inoculated with the siderophore and IAA-producing bacterial strain PVr_9, which shows ACC deaminase activity; (iv) plants inoculated with the siderophore and IAA-producing bacterial strain PVr_5, which shows ACC deaminase activity and contains the Bacillus *arsC* gene required for the reduction of As(V) to As(III); and (v) plants inoculated with the PVr_15 strain containing the Bacillus *arsC* gene. Fifty-milliliter aliquots of bacterial suspensions were individually added to the plants at the stem–soil interfacial area, whereas the same amount of sterile distilled H_2_O was added for non-inoculated control (CTR) plants. All pots, with four replicates per treatment, were alternately watered, two times a week, with an equal volume of sterile H_2_O. 

The experiment lasted 4 months and the inoculums were applied either at the beginning of the experiment or after 2 months [10]. 

At the end of the experiment, in order to determine As content, the plants were gently removed from the pots, washed three times with deionized H_2_O, and dissected into hypogeal (root) and epigeal (frond) portions and dried for the measurement of As content; soil samples were collected from each pot.

The As bioaccumulation factor (BAF) was calculated as indicated by Antenozio et al., 2021 [11], as a ratio of As concentration in fronds (frond BAF) or roots (root BAF), and the corresponding bioavailable As concentration in soil. The translocation factor (TF) was calculated as the root/shoot ratio [46].

For the experiment using sterilized Bagnaccio soil, the soil was sterilized by autoclaving at 121 °C for 15 min before use [10], and then plants were either not inoculated (CTR plants) or inoculated with all six bacterial strains (PVr_2, PVr_5, PVr_9, PVr_15, PVr_16, PVr_17) resistant to 100 mM As(V) as explained above.

### 4.4. Total Arsenic Determination

About 0.1 g of dried leaves, 0.3 gr of dried roots, and 0.5 gr of soils, rehydrated with 1 mL of ultrapure grade water (0.05 µS cm^−1^, Purelab ULTRA Elga, High Wycombe, UK), were mineralized in duplicate with 2 mL (for leaves and roots) and 5 mL (for soils) of 69% *v*/*v* ultrapure HNO_3_ (UltrexTM II, J.T. BakerTM, Avantor, Allentown, PA, USA) through a high-pressure microwave-assisted digestion system (Ultrawave, Milestone s.r.l., Sorisole, Italy). The operating conditions for leaf and root mineralization were previously described [47]. Soils were digested according to the EPA 3051A (2007) method, using a mineralization cycle with the following conditions: 1500 W, 120 bar, and 175 °C for 20 min, including heating and temperature holding. The digested samples, opportunely diluted with ultrapure grade water and filtered on 0.45 μm filters (Millex^®^-HA, Millipore, Merck, Darmstadt, Germany), were introduced into an ICP-OES spectrometer (Vista-MPX, Varian, Agilent Technologies, Santa Clara, CA, USA) with the configuration previously described [32]. The concentrations of total As (234.984 nm) were quantified using an external 7-point calibration line (0–50 mg L^−1^) prepared from a 1000 mg/L mono-element standard solution of As (TraceCERT^®^ Fluka Analytical, Sigma-Aldrich, St. Louis, MI, USA).

### 4.5. Plant and Data Analysis

Experiments were carried out using four (or five)-replicate samples and the values presented are expressed as means ± standard error (SE). The root length (cm) and root area (cm^2^) of *P. vittata* plants were measured using the analysis software ImageJ 1.53t (https://imagej.nih.gov/ij/ accessed on 1 May 2022). Differences among treatments were analyzed by using two-tailed and one-tailed Student’s *t* tests. Significant differences were defined when *p*-values were <0.05. PCA was performed with SPSS software (IBM SPSS Statistic, V22.0 accessed on 18 July 2024) verifying the absence of outliers, testing the sampling adequacy based on Kaiser–Meyrt–Olkin values (KMO = 0.714) and variable correlation by Bartlett’s test of sphericity (*p* < 0.05). 

## 5. Conclusions

**Figure 8:**
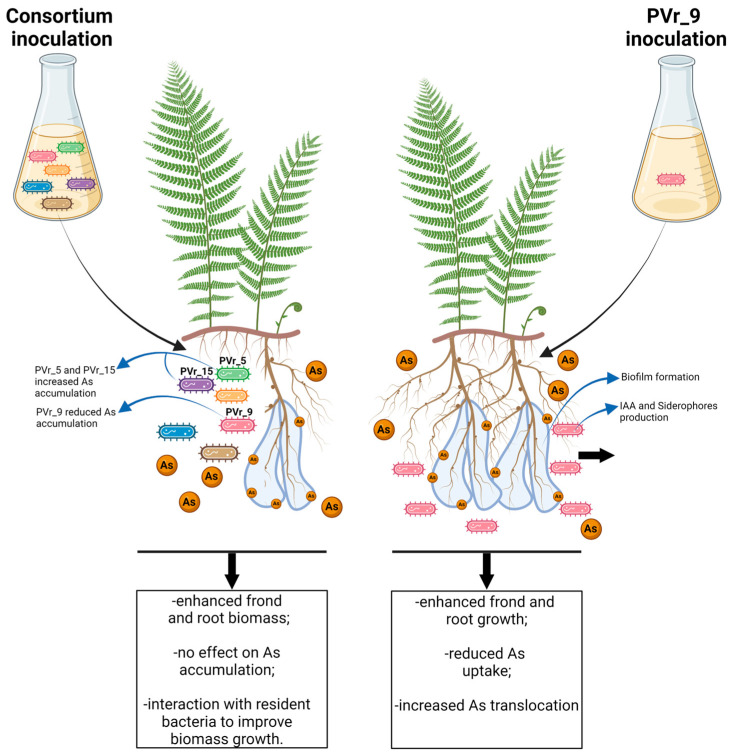
Schematic diagram shows plant growth and As uptake and translocation in *Pteris vittata* plants inoculated with the consortium of six As-resistant bacteria, or with PVr_9 strain alone.

In this study, we show that inoculums with a consortium of highly As-resistant bacteria isolated from the rhizosphere of *P. vittata* grown on Bagnaccio soil can increase fern growth on the same non-sterile soil, but has no effects on As phytoextraction (Figure 8). Our results clearly indicate that plant biomass enhancement requires the interaction of inoculated bacteria with resident microorganisms. Through single inoculations, we show that the PVr_9 strain, homologous to *A. radiobacter,* has the capacity to enhance plant growth and reduce As accumulation while increasing its translocation to the shoot (Figure 8). In contrast, the PVr_5 and PVr_15 isolates, classified as *P. urefaciens* and *A. schindleri*, substantially increase phytoextraction efficiency but induce a limited increase in plant growth. This clearly shows that plant growth and phytoextraction efficiency are not directly associated. To our knowledge, PVr_9’s characteristics have not been described previously, and future work is necessary to assess whether it can be used to confer As tolerance and reduce the content of As in cultivated species. 

On the other hand, strains PVr_5 and PVr_15 can be useful for phytoremediation experiments.

## Data Availability

The data are all presented in the paper and in the Supplementary Materials.

## Supplementary Materials

The following supporting information can be downloaded at https://www.mdpi.com/article/10.3390/plants13152030/s1: Table S1: Effect of bacterial inoculations with the consortium in *P. vittata* plants grown on non-sterile soil on translocation factor (TF) and on the bioconcentration factor of fronds and roots (frond BAF root BAF, respectively); Table S2: Effect of bacterial inoculations with the consortium in *P. vittata* plants grown on sterile soil on translocation factor (TF) and on the bioconcentration factor of fronds and root (frond BAF and root BAF, respectively); Table S3: Effect of bacterial inoculations with different isolates (PVr_9, PVr_5, and PVr_15) on translocation factor (TF), and on the bioconcentration factor of fronds and roots (frond BAF and root BAF, respectively).
