# Supplementary material for: Enhanced Growth and Contrasting Effects on Arsenic Phytoextraction in Pteris vittata through Rhizosphere Bacterial Inoculations
Source: Plants (Basel). 2024 Jul 24;13(15):2030. doi: 10.3390/plants13152030 (PMC11314128; doi:10.3390/plants13152030)
Supplement: Supplementary file 1 [file plants-13-02030-s001.zip › plants-3098844-supplementary.pdf]

Supplementary material

Table S1. Effect of bacterial inoculations with the consortium in *P. vittata* plants grown on non-sterile soil, on translocation factor (TF) and on the bioconcentration factor of fronds and roots (BAF-frond and BAF-root, respectively).

	**TF**	**BAF-frond**	**BAF-root**
**CTR plants**	12.41	15.75	1.27
**Inoculated plants**	11.96	13.72	1.15

Table S2. Effect of bacterial inoculations with the consortium in *P. vittata* plants grown on sterile soil, on translocation factor (TF) and on the bioconcentration factor of fronds and root (BAF-frond and BAF-root, respectively).

	**TF**	**BAF-frond**	**BAF-root**
**CTR plants**	11.08	17.63	1.59
**Inoculated plants**	10.35	6.75	0.65

Table S3. Effect of bacterial inoculations with different isolates (PVr_9, PVr_5 and PVr_15) on translocation factor (TF), and on the bioconcentration factor of fronds and roots (BAF-frond and BAF-root, respectively).

	**TF**	**BAF-frond**	**BAF-root**
**CTR plants**	4.80	9.62	2.01
**PVr_9 inoculated plants**	12.44	11.46	0.92
**PVr_5 inoculated plants**	4.62	17.09	3.70
**PVr_15 inoculated plants**	8.35	17.68	2.12
